# A truncating *Aspm* allele leads to a complex cognitive phenotype and region-specific reductions in parvalbuminergic neurons

**DOI:** 10.1038/s41398-020-0686-0

**Published:** 2020-02-13

**Authors:** Lillian Garrett, Yoon Jeung Chang, Kristina M. Niedermeier, Tamara Heermann, Wolfgang Enard, Helmut Fuchs, Valerie Gailus-Durner, Martin Hrabě de Angelis, Wieland B. Huttner, Wolfgang Wurst, Sabine M. Hölter

**Affiliations:** 1 Institute of Developmental Genetics, Helmholtz Zentrum München, Neuherberg, Germany; 2 German Mouse Clinic, Helmholtz Zentrum München, Neuherberg, Germany; 3 Max Planck Institute of Molecular Cell Biology and Genetics, Dresden, Germany; 4 Anthropology and Human Genomics, Department of Biology II, Ludwig Maximilians University Munich, Martinsried, Germany; 5 Institute of Experimental Genetics and the Helmholtz Zentrum München, Neuherberg, Germany; 6 Chair of Experimental Genetics, Faculty of Life and Food Sciences Weihenstephan, Technische Universität München, Freising-Weihenstephan, Germany; 7 German Center for Diabetes Research (DZD), Neuherberg, Germany; 8 Chair of Developmental Genetics, Faculty of Life and Food Sciences Weihenstephan, Technische Universität München, Freising-Weihenstephan, Germany; 9 Deutsches Zentrum für Neurodegenerative Erkrankungen e. V. (DZNE), München, Germany; 10 Munich Cluster for Systems Neurology (SyNergy), Adolf-Butenandt-Institut, Ludwig-Maximilians-Universität, München, Germany

**Keywords:** Molecular neuroscience, Autism spectrum disorders

## Abstract

Neurodevelopmental disorders are heterogeneous and identifying shared genetic aetiologies and converging signalling pathways affected could improve disease diagnosis and treatment. Truncating mutations of the abnormal spindle-like microcephaly associated (*ASPM*) gene cause autosomal recessive primary microcephaly (MCPH) in humans. ASPM is a positive regulator of Wnt/β-Catenin signalling and controls symmetric to asymmetric cell division. This process balances neural progenitor proliferation with differentiation during embryogenesis, the malfunction of which could interfere with normal brain development. *ASPM* mutations may play a role also in other neurodevelopmental disorders, nevertheless, we lack the details of how or to what extent. We therefore assessed neurodevelopmental disease and circuit endophenotypes in mice with a truncating *Aspm*^*1–7*^ mutation. *Aspm*^*1–7*^ mice exhibited impaired short- and long-term object recognition memory and markedly enhanced place learning in the IntelliCage®. This behaviour pattern is reminiscent of a cognitive phenotype seen in mouse models and patients with a rare form of autism spectrum disorder (ASD) as well as in mouse models of altered Wnt signalling. These alterations were accompanied by ventriculomegaly, corpus callosum dysgenesis and decreased parvalbumin (PV)+ interneuron numbers in the hippocampal Cornu Ammonis (CA) region and thalamic reticular nucleus (TRN). PV+ cell number correlated to object recognition (CA and TRN) and place learning (TRN). This opens the possibility that, as well as causing MCPH, mutant *ASPM* potentially contributes to other neurodevelopmental disorders such as ASD through altered parvalbuminergic interneuron development affecting cognitive behaviour. These findings provide important information for understanding the genetic overlap and improved treatment of neurodevelopmental disorders associated with ASPM.

## Introduction

The overlap in symptoms between neurodevelopmental disorders (NDDs) suggests that there is at least partial overlap in genetic aetiology and affected signalling networks during brain development. Therefore, the challenge is to understand better the consequences of altered gene expression, shared genetic aetiology and convergent signalling pathways to account for clinical variation and ultimately to improve disease diagnosis, treatment and prevention^1^. Abnormal spindle-like microcephaly associated (*ASPM*) truncating mutations are the most common cause of the NDD autosomal recessive primary microcephaly (MCPH) in humans^2^. This is a rare, genetically heterogeneous, disease where patients exhibit a smaller, albeit structurally normal, brain closely linked with intellectual disability. Limited association study data imply an *ASPM* contribution also to the pathogenesis of other NDDs including schizophrenia^3^, communication disorder^4^ and ASD^5,6^. Nevertheless, this has not been reinforced by additional patient data, validated empirically nor is it clear through what mechanism *ASPM* could contribute to features of these NDDs.

The *ASPM* gene is located on chromosome 1q31, composed of 28 exons and encodes 3477 amino acids^2^. In mice, it is expressed in the ventricular zone of the neocortex during embryonic neurogenesis where it localises to the mitotic spindle poles and midbody^2,7^. There, it controls symmetric to asymmetric cell division, which is important for balancing neural progenitor proliferation with differentiation^8^. ASPM is also essential for correct neuronal migration during corticogenesis^9^ and was positively selected during primate evolution where it contributed to the genetic basis of brain size^10^. Recent evidence indicates that ASPM is a positive regulator of the canonical Wnt/β-catenin signal transduction pathway and overexpression of β-catenin can rescue defective neurogenesis induced by ASPM reduction in mice^9^. *Aspm* is also expressed in the adult brain^11,12^. While there is correlational data potentially implicating ASPM in adult neurogenesis^11^, an exact function remains unclear.

Previously, a mutant mouse line, *Aspm*^*1–7*^, was generated by gene-trap vector insertion into the intron between exon 7 and 8 producing a MCPH patient-like protein truncation^13^. The majority of human microcephaly patients with *ASPM* mutations have protein truncations in or before the region encoded by exon 26^13,14^. The truncated protein consists of only the microtubule binding domain and lacks the C-terminal amino acids, the calponin homology domains and the calmodulin-binding isoleucine-glutamine (IQ) repeats. This allele, believed to be loss-of-function, causes mild microcephaly in mice where brain size was ~90% that of controls. Currently, there is only limited information detailing the cognitive alterations consequent to *Aspm* disruption. A recent report undertook neuropsychologic assessment of MCPH patients with *ASPM* mutations and revealed mild-to-moderate intellectual disabilities^15^. They nevertheless observed 50% or more reductions in the surface area of cortical regions, the relative magnitude of which was not observed previously in the *Aspm*^*1–7*^ mice. The aim of this study was thus to assess the causal relationship between this allele, a concomitant mild form of microcephaly and changes in mouse cognitive ability as well as neuronal cell populations. By applying a disease-relevant endophenotype strategy, i.e., focusing on objectively quantifiable components of gene-to-behaviour-to-neural circuit pathways^16^, we wanted also to ascertain whether *Aspm* alterations have relevance for other NDDs. We therefore applied a comprehensive analysis that addressed multiple behaviour modalities including aspects of emotionality, social affinity/memory, working, recognition and spatial memory. Given the established *Aspm* influence on embryonic neurogenesis, we endeavoured to understand the effect of this mutation on adult neurogenesis. As well as quantifying this process, we implemented running wheel exercise, a known inducer of adult hippocampal neurogenesis, to assess potential amelioration of genotypic behavioural effects with this environmental enrichment^17^.

## Materials and methods

### Animals

**Fig. 1:**
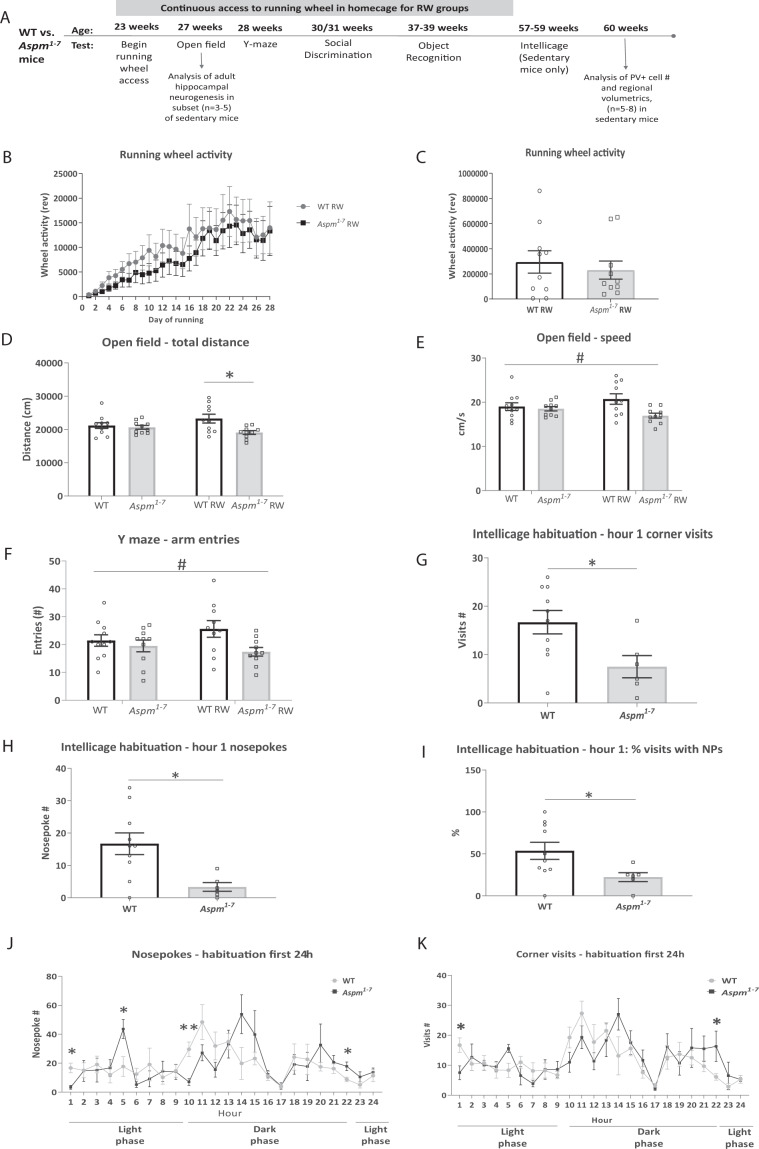
Blunted spontaneous reactions to a novel environment in *Aspm*^*1–7*^ mice. Timeline and sequence of tests performed on *Aspm*^*1–7*^ mice and age at testing (**a**). There were no genotypic differences in either running wheel activity on each day over first 28 days (**b**) or in total over first 28 days *n* = 10 WT, *n* = 10 *Aspm*^*1–7*^ mice (**c**). Locomotor activity (total distance, **d**) and movement speed (**e**) during the 20-minute open field test. Locomotor activity (arm entries) during the 5-minute Y-maze test of spontaneous alternations (**f**). Corner visits (**g**), nosepokes (**h**) and % corner visits with nosepokes (NPs) (**i**) during the first hour of habituation to the novel IntelliCage® environment. Nosepokes (**j**) and corner visits (**k**) during the first 24 h of habituation to the IntelliCage®. *p < 0.05, **p < 0.01 WT vs *Aspm*^*1–7*^ mice. ^#^*p* < 0.05 main effect of genotype with 2-way ANOVA (WT vs. *Aspm*^*1–7*^ mice independent of exercise experience). Data are means ± SEM. WT = wildtype control mice, RW = running wheel, PV + = parvalbumin +, rev = wheel revolutions.

All tests performed were approved for the ethical treatment of animals by the responsible authority of the Regierung von Oberbayern (Government of Upper Bavaria). The *Aspm*^*1–7*^ gene trap mutant mouse line was produced as described in detail previously^13^. It was generated from ES cells [AA0137, AspmGt (AA0137) Wtsi; vector pGT0lxr; obtained from the Sanger Institute Gene Trap Resource] by blastocyst injection and chimeras that had germ line transmission were crossed to C57BL/6JOlaHsd mice for more than 10 backcross generations. For the AA0137 ES cell line, PCR and sequencing of genomic DNA (forward primer: targeting the upstream exon, reverse primer: targeting the 5′ end of β-geo) were used to show that the insertion site for the vector pGT01xr was in the intron between exon 7 and 8, 1386 nt downstream from the intron start site. In this initial validation of the line, RT-PCR and immunohistochemistry were used to establish the expression of the truncated ASPM protein tagged with lacZ. RT-PCR was performed on embryonic day (E) 13.5 whole embryo DNA to show that endogenous *Aspm* mRNA was lost in the mutant mice. The endogenous WT transcript was present only in the WTs and only the gene-trapped transcript was present in the homozygotes. Furthermore, to show that ASPM protein was tagged with β-geo, immunostaining with β-galactosidase (β-gal) together with ASPM on E10.5 dorsal telencephalon sections was performed. In homozygous mutant mice, β-gal and ASPM were colocalised at metaphase spindle poles whereas β-gal immunoreactivity was absent in the WT. This demonstrated that ASPM was tagged with β-geo. All experiments involved the use of male homozygous mutant mice and their littermate control wild types. The sample size used for the behavioural analyses was based on that used for similar published analyses. Mice were assessed from the age of 23 weeks according to the sequence and ages shown in Fig. 1a. After 4 weeks of voluntary running wheel access, mice (WT *n* = 11, *Aspm*^*1–7*^ *n* = 10, WT RW *n* = 10, *Aspm*^*1–7*^ RW *n* = 10) were assessed in a series of behavioural assays. Both mice with access to running wheels and sedentary controls from each group were tested in parallel. Only sedentary mice were tested in Intellicage® due to setup limitations in giving equal wheel access and activity quantification to all mice within this environment. We therefore focused our cell population quantifications on sedentary mice only. These analyses remain to be replicated in an independent cohort and no method of randomization was used for either behaviour or histological analyses. As mouse behaviour was analysed in a counter balanced design, the observer was not blind to the genotypes. More detailed methods information is contained in the Supplementary Material.

### Voluntary running wheel activity assessment

The home-cage voluntary running wheel behaviour of mutant (*Aspm*^*1–7*^ RW, *n* = 10) and wildtype control (WT RW, *n* = 10) mice was analysed as described previously using low profile wireless running wheels (RWs) (Wheel Manager Software, Med Associates Inc., VT, and USA)^18^. Mice were singly housed throughout the running period from age 23 to 37 weeks and individual running activity recorded for analysis.

### Behavioural assays

#### Open field

The Open Field (OF) analysis was as described before using the ActiMot system (TSE, Bad Homburg, Germany)^18^. The arena consisted of a transparent and infra-red light permeable acrylic test arena with a smooth floor (internal measurements: 45.5 × 45.5 × 39.5 cm) and illumination at 150 lux in the corners and 200 lux in the middle.

#### Y Maze

Spontaneous alternations, alternate arm returns and entries were assessed using the Y-Maze as reported previously^19,20^. The Y maze was composed of opaque light grey PVC and had three identical arms (30 × 5 × 15 cm) placed at 120° from each other. The illumination in the centre of the maze was 100 lux.

#### Social discrimination

The Social Discrimination procedure involved using ovariectomised 129Sv stimulus females as shown previously^19,21^. Measures included time spent in social investigation (“social affinity”) and a social recognition index (“social memory”).

#### Short and long-term object recognition memory

The object recognition procedure was as described previously^20^. The recognition index is the percentage time spent investigating the unfamiliar novel object/(time spent investigating familiar + unfamiliar objects).

#### Place and reversal learning and working memory in the IntelliCage®

The IntelliCage® (NewBehavior, TSE Systems GmbH, Bad Homburg, Germany) assessed place and reversal learning and working memory (“Patrolling”) (detailed procedure in ref. ^20^). It is an automated behavioural analysis apparatus consisting of a relatively large plastic homecage (55 × 37.5 × 20.5 cm^3^) where the four cage corners are operant conditioning chambers (15 × 15 × 21 cm^3^).

#### Tissue preparation

Fixed and cryoprotected mouse brains of 60 week old mice were sectioned in a rostro-caudal direction on a dry ice-cooled block with a sliding microtome (Leica, Bensheim) as detailed previously^22^. A one-in-six series of 40 µm-thick coronal free-floating sections was taken for analysis.

#### Immunostaining

For immunostaining of doublecortin (DCX)+ and parvalbumin (PV)+ cells, an Avidin-Biotin Complex ABC method like that employed previously^18,22^ was used. The materials used were polyclonal anti-DCX antibody (1:1000, Catalog #: Ab18723, Abcam) with a biotinylated goat anti-rabbit IgG (1:300; Biotin-SP AffiniPure Goat Anti-Rabbit IgG, Jackson ImmunoResearch Inc, USA) and a primary monoclonal mouse anti-PV antibody (1:1000, Catalog #: PV235, SWANT, Switzerland) with a biotinylated rabbit anti-mouse IgG (1:300, Biotin-SP AffiniPure Rabbit Anti-Mouse IgG, Jackson ImmunoResearch Inc, USA). An ABC complex was prepared according to manufacturers instructions (VECTASTAIN Elite ABC HRP Kit PK-6100, VECTOR LABORATORIES, INC., Burlingame, USA). Negative controls, with omission of the primary antibodies, revealed no positive staining.

#### Unbiased stereological estimates of DCX+ and PV+ cell numbers and volumetrics

DCX+ and PV+ cell numbers were estimated in specific regions of interest (ROIs) with design-based stereology using the Stereo Investigator software system (StereoInvestigator, MBF Biosciences Inc.) on every sixth serial 40 µm coronal section with the Optical Fractionator probe as described previously^22,23^. The observer was blind to the experimental groups during analysis. When tissue from an animal was damaged during processing, it was excluded from the analysis. A subset of younger (27 weeks old) sedentary WT and *Aspm*^*1–7*^ mice (*n* = 3–4) were sacrificed and doublecortin (DCX)+ cell number (to index adult hippocampal neurogenesis) was analysed at a timepoint that coincided with the start of the behaviour testing sequence. DCX+ cells were estimated within the dorsal hippocampal dentate gyrus. The subgranular zone of the hippocampal dentate gyrus was selected for analysis because it is one of two main neurogenic niches in the adult mouse brain and has the potential to play a role in spatial memory^24^. PV+ cell numbers were estimated within brain regions known to be involved in cognitive behaviour and/or dysfunctional in NDDs. These included the hippocampal dentate gyrus, Cornu Ammonis (CA) 1, 2/3 regions and the anterior cingulate cortex (ACC). We also quantified the PV+ cell number in the thalamic reticular nucleus (TRN) because of the dense PV+ cell population here and our previous evidence that it may play a role in NDD endophenotypes^23^. Volumetric analysis of selected ROIs was performed using the Cavalieri estimator probe as described previously^22^. The following brain ROIs were analysed: corpus callosum, lateral ventricles, CA1, 2, 3 and DG.

#### Statistics

Data was analysed using Shapiro-Wilk test for normal distribution and then analysed with two-way ANOVA (with post hoc Tukey’s to test genotype-exercise interaction effects for open field, Y maze, social discrimination and object recognition) and repeated measures ANOVA (with post hoc Sidak’s for Intellicage data); unpaired Student’s *t*-test (for DCX+ and PV+ cell population analyses, total running wheel distance during the first 4 weeks of running, IntelliCage® habituation phase nosepokes, corner visits and % visits with nosepokes); Grubb’s test (to detect and exclude statistical outliers – one sedentary homozygous mutant mouse was identified as an outlier and excluded from long-term object recognition memory analysis) and Pearson’s correlation test using GraphPad Prism version 7.03 for Windows (GraphPad Software, La Jolla, California, USA, www.graphpad.com). For all tests, a *P* value < 0.05 was considered significant and data are presented as means ± SEM. A correction for multiple testing was not performed.

## Results

### A truncating *Aspm* mutation blunts novelty-induced activity responses

In order to assess the voluntary exercise behaviour consequent to *Aspm* disruption, we gave mutant mice and littermate controls access to running wheels within their homecage. The *Aspm*^*1–7*^ mice showed normal voluntary wheel running activity (Student’s *t*-test: *t*(18) = 0.56, n.s., Supplementary Table 2). The amount of running wheel activity per 24 h period as well as the total over the course of the first 4 weeks did not differ between the groups (Fig. 1b, c). Therefore, mutants do not differ from controls in terms of voluntary exercise behaviour.

In the open field (see also Supplementary Table 1), a test of spontaneous reactions to a novel mildly stressful environment, there was a significant decrease in locomotor activity and speed in the mutant mice (two-way ANOVA, genotype effect total distance: *F*(1,37) = 7.14, *p* = 0.01, genotype effect velocity: *F*(1,37) = 6.56, *p* = 0.02, Fig. 1d, e). Furthermore, the locomotor difference effect was more pronounced in the mutant mice with access to a running wheel (two-way ANOVA, distance travelled genotype × exercise interaction effect: *F*(1,37) = 4.24, *p* = 0.047, post hoc Tukey’s test, WT RW vs. *Aspm*^*1–7*^ RW, *p* = 0.01). The mutant mice also showed decreased arm entries in the Y maze (two-way ANOVA, genotype effect: *F*(1,37) = 5.18, *p* = 0.03, Fig. 1f) as well as decreased corner visits, nosepokes and % corner visits with nosepokes during the first hour on introduction into the IntelliCage® (Unpaired *t*-test corner visits: *t*(14) = 2.55, *p* = 0.02, nosepokes: *t*(14) = 2.97, *p* = 0.01, % visits with nosepokes: *t*(14) = 2.25, *p* = 0.04, Fig. 1g–k Supplementary Table 2). They also reduced nosepoke number when lights were switched off at the beginning of the active phase of the cycle (Unpaired *t*-test nosepokes during hour after lights off: *t*(12) = 3.67, *p* = 0.003, Fig. 1j). Thus, overall, mutant mice exhibit hypoactive responses to environmental changes.

### A truncating *Aspm* mutation impairs object recognition memory

**Fig. 2:**
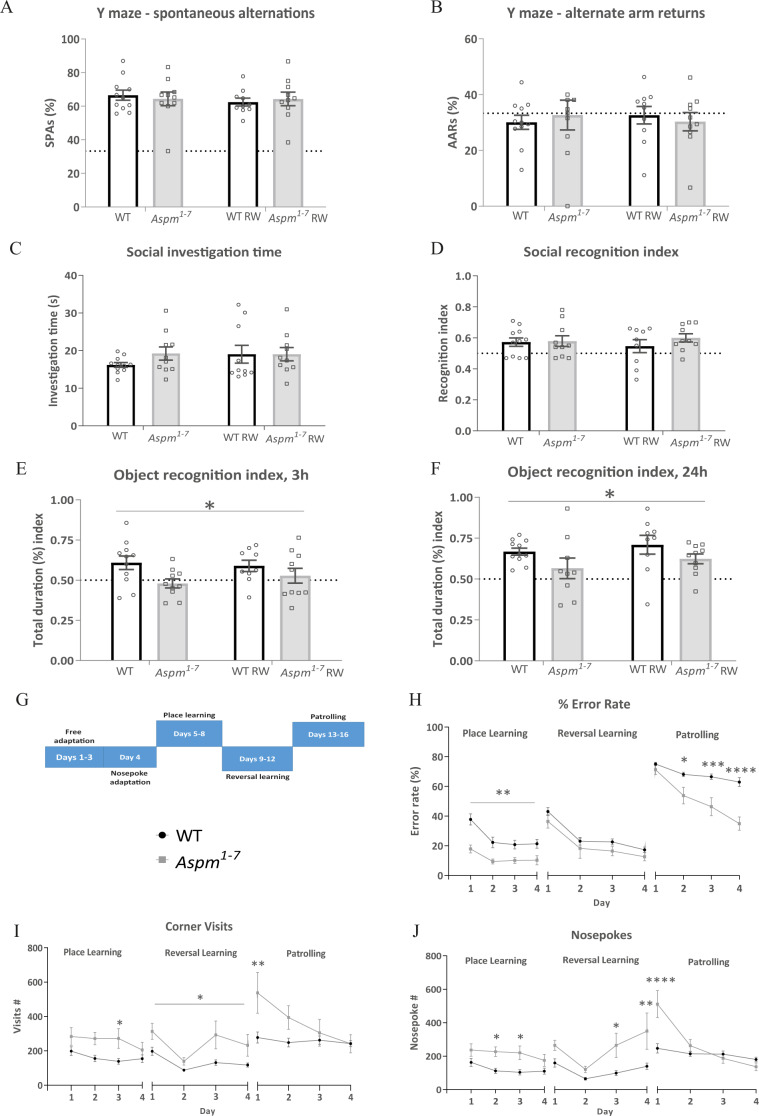
Impaired short- and long-term object recognition memory in the *Aspm*^*1–7*^ mice. The number of spontaneous alternations (SPAs, **a**) and alternate arm returns (AARs, **b**) in the Y-maze. Dotted line indicates 33% chance level. Social investigation time during the sample phase (**c**) and recognition index (**d**) during the social discrimination test. Dotted line indicates 50% chance level. The short-term 3 h (**e**) and long-term 24 h (**f**) recognition index from the object recognition test. *p < 0.05 WT vs. *Aspm*^*1–7*^ mice, two-way ANOVA genotype effect. The IntelliCage® place and reversal learning and working memory (patrolling) protocol overview used for the *Aspm*^*1–7*^ mice is shown in (**g**). During the place learning and patrolling phases, the *Aspm*^*1–7*^ mice showed enhanced place learning and working memory as indexed by a decreased % error rate (**h**). *p < 0.05, **p < 0.01, ***p < 0.01, ****p < 0.0001 WT vs. *Aspm*^*1–7*^ mice, repeated measures ANOVA with post hoc Sidak’s test. The *Aspm*^*1–7*^ mice engaged in an increased number of corner visits (**i**) and nosepokes (**j**) during the place and reversal learning and patrolling phases. *p < 0.05, **p < 0.01, ****p < 0.0001 WT vs. *Aspm*^*1–7*^ mice, repeated measures ANOVA with post hoc Sidak’s test. WT *n* = 9, *Aspm*^*1–7*^ *n* = 6. Data are means ± SEM.

To understand the cognitive ability of the mutant mice, we applied a series of learning and memory assays. In the Y-maze, a test of simple working memory, the truncating *Aspm* mutation did not alter the number of spontaneous alternations (two-way ANOVA genotype effect: *F*(1,37) = 0.002, *p* = 0.96, Fig. 2a) or alternate arm returns (two-way ANOVA genotype effect: *F*(1,37) = 0.001, *p* = 0.97, Fig. 2b). Voluntary exercise also did not affect Y-maze spontaneous alternation behaviour (two-way ANOVA, exercise effect: *F*(1,37) = 0.36, *p* = 0.55, Supplementary Table 1). Furthermore, there were no genotype effects on social affinity in the social discrimination test (two-way ANOVA genotype effect: *F*(1,37) = 0.79, *p* = 0.38, Fig. 2c) or on the recognition index (two-way ANOVA genotype effect: *F*(1,37) = 0.84, *p* = 0.37, Fig. 2d). Voluntary running wheel access did not affect social memory ability or social affinity (recognition index: two-way ANOVA exercise effect: *F*(1,37) = 0.006, *p* = 0.94; social affinity: two-way ANOVA exercise effect: *F*(1,37) = 0.61, *p* = 0.44).

While neither of these forms of memory were markedly affected by the mutation, from the age of 37 weeks, the mutant mice showed impaired short- and long-term object recognition memory. Here the recognition memory index decreased after both 3 and 24 h (two-way ANOVA, genotype effect short-term memory: *F*(1,36) = 5.94, *p* = 0.02, genotype effect long-term memory: *F*(1,35) = 4.57, *p* = 0.04, Fig. 2e, f, Supplementary Table 1). Engaging in exercise did not alter either short- or long-term recognition memory (two-way ANOVA, exercise effect short-term memory: *F*(1,36) = 0.14, *p* = 0.71, exercise effect long-term memory: *F*(1,35) = 1.30, *p* = 0.26). This truncating *Aspm* mutation therefore causes specific recognition deficits.

### A truncating *Aspm* mutation enhances place learning and working memory ability

Using the IntelliCage® apparatus, place and reversal learning ability were assessed in these mice. During the IntelliCage® place learning trial (Fig. 2g for protocol overview), the *Aspm*^*1–7*^ mice showed decreased % error rate compared to control mice (RM ANOVA genotype effect: *F*(1,13) = 12.52, *p* = 0.004, day effect: *F*(3,39) = 17.88, *p* < 0.0001, interaction effect: *F*(3,39) = 2.25, *p* = 0.10, Fig. 2h). As an index of activity during this trial, *Aspm*^*1–7*^ mice made slightly more corner visits (Fig. 2i). Nevertheless, this effect was only significant during the third day of the place learning trial (RM ANOVA interaction effect: *F*(3,39) = 5.03, *p* = 0.005, post hoc Sidak’s test on day 3 *t*(52) = 2.86, *p* = 0.02). The mutant mice made also more nosepokes, an effect that was significant during day 2 and 3 of the place learning (RM ANOVA interaction effect: *F*(3,39) = 3.65, *p* = 0.02, post hoc Sidak’s test for day 2: *t*(52) = 3.08, *p* = 0.01, day 3: *t*(52) = 3.14, *p* = 0.01, Fig. 2j).

There were no genotype effects on behavioural flexibility in the reversal learning phase (RM ANOVA genotype effect: *F*(1,13) = 2.53, *p* = 0.14, Fig. 2h). Concerning activity during this testing phase, the truncating *Aspm* mutation led to increased corner activity as indexed by an increased number of corner visits by the mutant mice (RM ANOVA genotype effect: *F*(1,13) = 6.21, *p* = 0.03, Fig. 2i). The *Aspm*^*1–7*^ mutation also led to a significantly increased number of nosepokes during day 3 and 4 of the reversal learning phase (RM ANOVA interaction effect: *F*(3,39) = 2.99, *p* = 0.04, post hoc Sidak’s test day 3 wt vs. hom *t*(52) = 2.83, *p* = 0.03, day 4 wt vs. hom *t*(52) = 3.58, *p* = 0.003, Fig. 2j).

From day 2 to day 4 of patrolling, the mutant mice exhibited a progressively lower % error rate compared to controls (RM ANOVA interaction effect: *F*(3,39) = 7.48, *p* = 0.0005, post hoc Sidak’s test day 2 wt vs. hom: *t*(52) = 3.02, *p* = 0.02, day 3 wt vs. hom: *t*(52) = 4.26, *p* = 0.0003, day 4 wt vs. hom: *t*(52) = 5.93, *p* < 0.0001, Fig. 2h). The truncating *Aspm* mutation was associated with a clear improvement (decreased % error rate) between day 1 and day 2 of patrolling (post hoc Sidak’s day 1 vs. day 2 in homs *t*(39) = 4.27, *p* = 0.0007, day 3 vs. day 4 *t*(39) = 2.80, *p* = 0.047). There was no such improvement in the WT mice. With respect to activity during this testing phase, the truncating *Aspm*^*1–7*^ mutation led to an increased number of corner visits during day 1 of patrolling without significant differences during the other three days (RM ANOVA interaction effect: *F*(3,39) = 13.41, *p* < 0.0001, post hoc Sidak’s test day 1 wt vs. homs *t*(52) = 3.53, *p* = 0.004, Fig. 2i). Likewise, the *Aspm*^*1–7*^ mutant mice executed significantly more nosepokes on the first day of patrolling; a pattern of activity not visible during the other three days of this task (RM ANOVA interaction effect: *F*(3,39) = 24.11, *p* < 0.0001, post hoc Sidak’s test, day 1 wt vs. hom *t*(52) = 5.78, *p* < 0.0001, Fig. 2j). Thus, the truncating *Aspm* mutation leads to clearly enhanced place learning as an index of spatial memory and patrolling ability as an index of working memory.

### *Aspm* mutation causes regional decreases in PV+ fast-spiking inhibitory interneurons

**Fig. 3:**
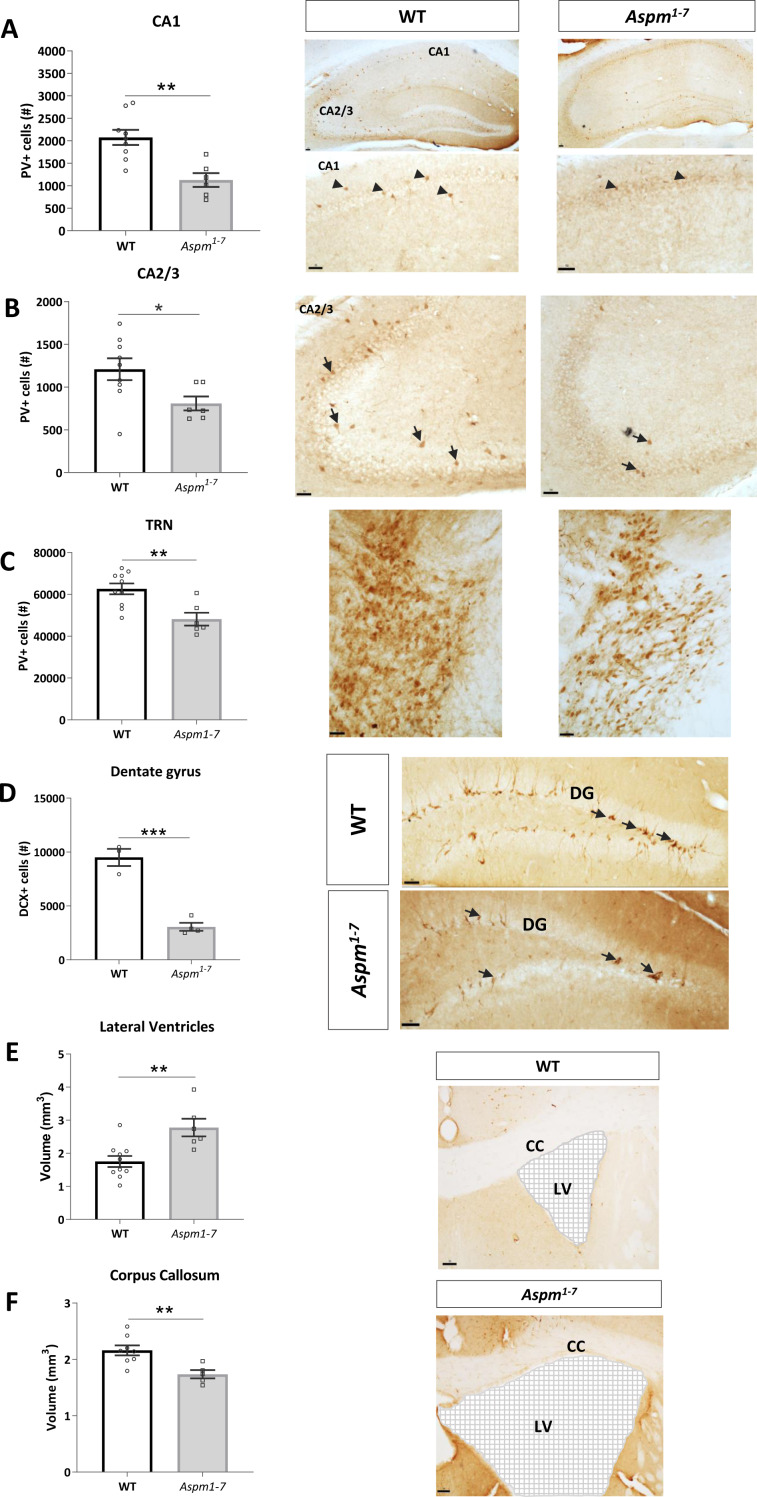
Decreased number of fast-spiking parvalbumin (PV)+ interneuron numbers. Estimates of the number of PV+ interneurons revealed decreases in both the Cornu Ammonis (CA) 1 (**a**) and 2/3 (**b**) regions of the hippocampus. A similar decreased PV+ cell number was detected in the thalamic reticular nucleus (TRN) in *Aspm*^*1–7*^ mutant mice compared to wild-type (WT) controls (**c**). *p < 0.05, **p < 0.01 WT vs *Aspm*^*1–7*^ mice, unpaired Student^’^s *t*-test. Scale bar = 50 µm. Arrows on photomicrographs point to PV+ cells. Estimates of total dorsal dentate gyrus doublecortin (DCX)+ cell number revealed decreases in the *Aspm*^*1–7*^ mice compared to wild-type (WT) controls. Arrows on photomicrographs reveal DCX+ cells (**d**). Cavalieri volumetric estimates revealed increased lateral ventricle volume (**e**) and decreased corpus callosum volume (**f**) in the *Aspm*^*1–7*^ mice compared to WT controls. Grids highlight the larger area of the lateral ventricles. ***p < 0.001, **p < 0.01 WT vs *Aspm*^*1–7*^ mice, unpaired Student’s *t*-test. Scale bar = 50 µm. DG = dentate gyrus, LV = lateral ventricle, CC = corpus callosum.

The GABAergic inhibitory interneurons are a key cell population crucial for modulating local circuit activity in the hippocampus and cortex. Of these inhibitory interneurons, the subset of PV+ GABAergic neurons is known to play a role in cognition^25^. Therefore, we next assessed the effect of the truncating *Aspm* mutation on the number of PV+ fast-spiking inhibitory GABAergic interneurons. These cells are involved in perisomatic inhibition and innervate and modulate the excitability of populations of hippocampal principal cells and thereby maintain a normal excitation/inhibition (E/I) balance. To determine whether alterations in PV+ GABAergic interneurons formed the substrate for the enhanced place learning in these *Aspm*^*1–7*^ mice, we quantified the number of PV+ cells in the hippocampal CA1 and CA2/3 regions. The *Aspm* truncating mutation led to a clear significant decrease in the number of PV+ cells in the CA1 region (Unpaired *t*-test: *t*(13) = 3.92, *p* = 0.002, Fig. 3a, Supplementary Table 3). Furthermore, there was a decrease in the number of PV+ cells in the CA2/3 region of these animals (Unpaired *t* test: *t*(13) = 2.34, *p* = 0.04, Fig. 3b). To establish whether other NDD-associated brain areas were affected by the truncating *Aspm* mutation, we quantified the number of PV+ cells in the ACC, the hippocampal dentate gyrus and the TRN of the thalamus. While there was no clear genotype-related difference in either the ACC (Unpaired *t*-test: *t*(10) = 1.65, *p* = 0.13, Supplementary Table 3) or dentate gyrus (Unpaired *t*-test: *t*(10) = 0.004, *p* = 0.10, Supplementary Table 3), in the TRN, there was a significant decrease in the number of PV+ cells (Unpaired *t*-test: *t*(14) = 3.52, *p* = 0.003, Fig. 3c, Supplementary Table 3). Disrupted *Aspm* thus alters the number of PV+ cells in specific brain regions.

### *Aspm*^*1–7*^ causes impaired adult neurogenesis, ventriculomegaly and corpus callosum dysgenesis

It is known that *Aspm* is expressed in the proliferative zones of the developing mouse brain where it is necessary for maintaining the spindle pole of mitotic neuroepithelial cells in symmetric proliferation^7^. Furthermore, there was an upregulation of *Aspm* in the adult rat hippocampus with increased proliferation suggesting a role for ASPM in adult hippocampal neurogenesis^11^. We thus quantified the number of DCX+ cells in the dorsal dentate gyrus as a surrogate marker of adult neurogenesis. The dorsal dentate gyrus preferentially mediates cognitive function including pattern separation and spatial learning and memory^26,27^. There was a clear decrease in the number of DCX+ cells (*t*(5) = 8.13, *p* = 0.0005, Fig. 3d) indicating a profound decrease in adult hippocampal neurogenesis.

Given this reduction in adult hippocampal neurogenesis, we assessed whether there were volumetric differences that would be related to such an impairment. It was shown previously that young adult *Aspm*^*1–7*^ mutant mice exhibit a mild microcephaly analogous to human patients with a truncating *ASPM* mutation and a thinner neocortex^13^. Subcortical structures were not assessed in this study, thus we here focused our volumetric analysis firstly on the hippocampus. We established that there were no volumetric differences between the genotypes in any of the hippocampal subfields (Supplementary Table 3). We also performed a volumetric analysis of both the lateral ventricles and the corpus callosum as alterations in these brain areas have been shown in both patients and *Aspm* −/− mice^28–30^. Here we also determined that the lateral ventricles were significantly larger compared to controls (*t*(14) = 3.46, *p* = 0.004, Fig. 3e). Furthermore, the volume of the corpus callosum was clearly smaller in the *Aspm*^*1–7*^ mutant mice indicative of corpus callosum dysgenesis (*t*(11) = 3.33, *p* = 0.007, Fig. 3f).

### Correlation between cognitive and neuropathological alterations in *Aspm*^*1–7*^ mice

**Fig. 4:**
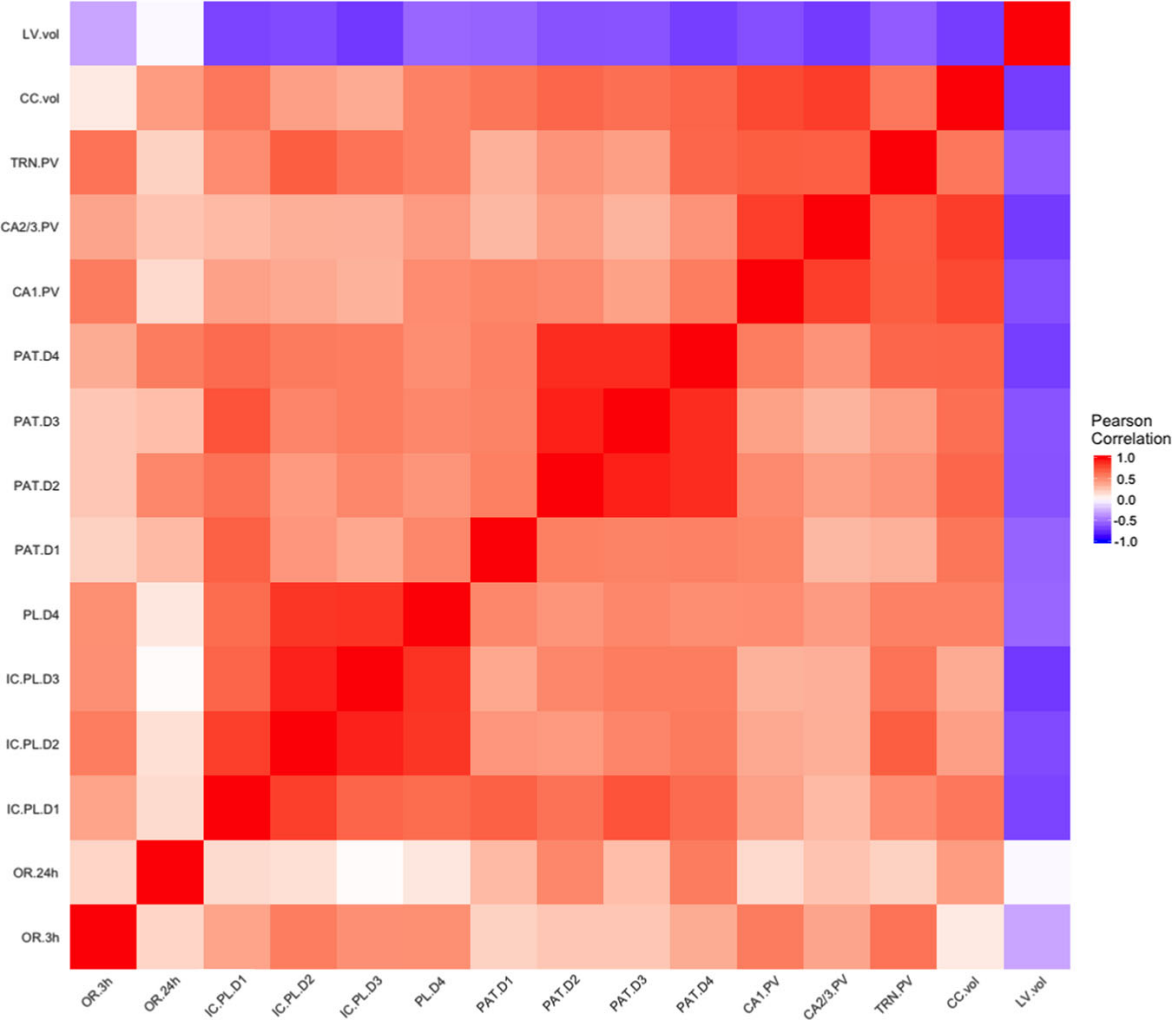
Correlation matrix of the altered behaviour and brain tissue parameters in all subjects. Correlations were obtained by calculating a Pearson’s correlation coefficient. Red is a positive correlation and blue is a negative correlation. LV vol = lateral ventricle volume, CC vol = corpus callosum volume, TRN = thalamic reticular nucleus, PV = parvalbumin+ cells, CA = Cornu Ammonis, PAT = patrolling in Intellicage, D = day, IC PL = place learning in Intellicage, OR = object recognition index.

To determine whether the altered object recognition, place learning and working memory ability in *Aspm*^*1–7*^ mice may be related to and/or a consequence of the altered PV+ cell number, ventriculomegaly and corpus callosum dysgenesis, we performed a Pearson’s correlation analysis. From the highlights of this analysis of all subjects focusing on the parameters that showed alterations in the *Aspm*^*1–7*^ mice (see Fig. 4 and Supplementary Table 4 for results), we observed better short-term (3 h) object recognition ability correlated with more PV+ cells in the TRN and the hippocampal CA1 region as was long-term (24 h) object recognition and PV+ cells in all three regions (TRN, CA1, CA2/3). Stronger short-term object recognition ability was also associated with impaired place learning (increased % error rate) albeit significant only during day 2. Notably, more TRN PV+ cells correlated with a higher % error rate in place learning and patrolling. The converse was observed between LV size and % error rate, inferring that larger LV volumes were associated with decreased place learning and patrolling % error rate. The number of PV+ cells in the TRN, CA1 and CA2/3 regions were positively correlated with each other and with corpus callosum size. LV size was negatively correlated with PV+ cells in the TRN, CA1, CA2/3 and with corpus callosum size.

## Discussion

*ASPM* plays a role in the pathoaetiology of microcephaly with potential involvement in other NDDs^3,5^. To probe this NDD link further, we assessed the effect of a mouse truncating *Aspm* allele on neurodevelopmental disease and circuit endophenotypes. The truncating *Aspm* mutation-induced complex cognitive phenotype and correlated neuropathological alterations have important implications not just for understanding MCPH but potentially also other NDDs. Impaired object recognition is likely due to changes in the perirhinal cortex that is necessary to confirm previously encountered objects and for short-term recognition memory. The hippocampus is essential for long-term recognition memory and receives inputs from the perirhinal cortex (reviewed by ref. ^31^). Spatial reference and working memory are also regarded as predominantly hippocampus-based, for both spatial disambiguation and using environmental cues to generate a cognitive map of a location^32,33^. During the place learning and patrolling trials in IntelliCage®, the *Aspm*^*1–7*^ mice performed better, exhibiting decreased error rates. Thus, it is likely that the truncating *Aspm* allele alters neurocircuits recruited for the execution of these cognitive tasks and this is supported by our neuropathological analysis results.

While dissociable effects on different memory modalities may seem counterintuitive, such a combination has been described in humans previously, for example, in rare high functioning autistic patients exhibiting superior cognitive and learning ability and performance in map-related spatial tasks^34^. Furthermore, this phenotypic pattern is present in ASD-related genetic models such as *Taok2* KO mice^35^ and to a degree in the *Shank1* KO mice^36^, neuroligin-3 R451C mutant mice^37,38^, *Scrib1* KO mice^39^ and *Lrfn2* KO mice^40^ (see Table 1 for phenotype summaries). A previous study indicates correlations between spatial learning in the water maze and place learning in IntelliCage® implying common underlying mechanisms involved^41^. In light of this evidence, Hung and co-workers^36^ even proposed improved spatial learning ability as an endophenotype for this rare form of ASD. Therefore, the truncating *ASPM* mutation may not just have relevance for MCPH but also other NDDs including particular ASD subtypes. The slight hypo activity exhibited by the *Aspm*^*1–7*^ mice on exposure to novel environments (open field, Y maze, IntelliCage) bolsters this assertion. A feature of certain NDDs including ASD and sensory processing disorders in humans include hypo- or hyper-reactivity to sensory stimuli^42–44^. While this locomotor change may represent an altered anxiety response to novelty, it could also signify that this truncating *Aspm* mutation blunts responses to sensory stimuli including novel situations. For reasons not currently appreciated and requiring further study, the experience of chronic exercise heightens this effect with *Aspm* mutation. The complex cognitive phenotype also phenocopies alterations in mice with conditional knockout of β-catenin (CTNNB1) in GABAergic PV+ interneurons^45^. CTNNB1 is a key regulator of the canonical Wnt signalling pathway pivotal to neurodevelopment. *Aspm*, in turn, is a positive regulator of this canonical Wnt/β-catenin pathway^9^. Thus, *ASPM* loss-of-function could lead to such a mixed cognitive phenotype via alterations in this pathway and cell population.

**Table 1:** Summary of ASD- and Wnt-signalling-related mouse lines with enhanced spatial memory.

Mouse line	Biological pathway affected	Locomotor phenotype	OR phenotype	Spatial memory phenotype	Social phenotype	Reference
Neuroligin (NRLG) 3^*R451C*^ KI mice *(On Sv129/C57BL/6 hybrid genetic background tested between 2–4 months of age)*	Synapse function (post-synaptic adhesion molecule)	No	n/a	Enhanced spatial memory in Morris water maze	Impaired	^37^
Neuroligin (NRLG) 3^*R451C*^ KI mice (*Backcrossed for 10 generations on 129S2/SvPasCrl genetic background aged 3.7–5.4 months at start of behaviour testing*)	Synapse function (post-synaptic adhesion molecule)	Hyperactivity in novel environment	n/a	Enhanced spatial memory in Morris water maze	Impaired	^38^
Shank (SHANK)1 KO mice (*Backcrossed on C57BL/6 genetic background for 6 generations aged 3–5 months at start of behaviour testing*)	Synaptic function (interacts with PSD-95)	Hypoactivity in novel environment	n/a	Enhanced spatial memory in eight-arm radial maze	n/a	^36^
Thousand-and-one amino acid kinase (TAOK)2 KO mice (*On C57BL/6J genetic background and tested between 2.5 and 4.5 months of age*)	MAPK pathway (affects dendrite growth)	Hyperactivity in novel environment	Impaired	Enhanced spatial memory in Water maze	Impaired	^35^
Leucine rich repeat and fibronectin type III domain containing (Lrfn)2 KO mice *(Backcrossed on C57BL/6J background for 6 generations aged between 2–6 months old)*	Synaptic function (interacts with PSD-95)	Increased wheel running	n/a	Enhanced spatial memory in Morris water maze	Impaired	^40^
Scribble1 (Scrib1^*crc*^) mice (*Aged 10–11 weeks at start of testing*)	Synaptic function (interacts with PSD)	No	n/a	Enhanced spatial memory in Morris water maze	Impaired	^39^
Beta catenin (CTNNB1) KO in PV+ cells (*Aged 8.5 weeks at start of testing*)	Wnt/beta-catenin signalling	No but elevated repetitive behaviours	Impaired	Enhanced spatial memory in Morris water maze	Impaired	^45^
Prickle (PRICKLE)2 (*Backcrossed on C57BL/6J genetic background for more than 10 generations and aged 8–12 weeks at start of testing*)	Wnt signalling regulates synaptic development	No	No	Enhanced spatial memory in Barnes maze	Impaired	^66^

*n/a* not analysed, *OR* object recognition, *KO* knock out, *KI* knock in, *PV* parvalbumin, *ASD* autism spectrum disorders.

Fast-spiking PV+ GABAergic interneurons inhibit and synchronise principal cells during network oscillations^25,46^. They are generated in the embryonic medial ganglionic eminence^47^ where *Aspm* is highly expressed at E14.5 and E16.5^48^. The evidence described here infers that disrupted ASPM may alter cognitive function by regulating PV+ cell development leading to an altered NDD-related endophenotype. It appears then that the TRN and hippocampal CA are ROIs for understanding IntelliCage® place learning as well as, by extrapolation, enhanced spatial memory in NDDs. The dense shell of PV+ cells of the TRN encapsulates thalamo-cortical neurons, relaying sensory information between thalamus and somatosensory cortex. It suppresses distracting sensory inputs during attentional processing mediating normal cognition^49,50^. It is possible that aberrant TRN PV+ cell number leads to thalamic excitation/inhibition imbalance modifying attentional capacity and affecting cognitive ability. Notably, correlational analysis revealed potential opposing roles of TRN PV+ cells in object recognition vs. place learning where a deficit is detrimental to the former yet advantageous for the latter. Further experiments are needed to address the fundamental mechanisms involved and to assess putative thalamocortical and hippocampal excitation/inhibition imbalances in detail. The possible contribution of CA1 PV+ interneurons to impaired object recognition is consistent with a previous observation^51^. How PV+ cell numbers decrease requires further study. Nevertheless, altered Wnt signalling is a likely explanation given that a similar complex cognitive phenotype occurs in mice with CTNNB1 KO in PV+ cells and the established role of ASPM in regulating this pathway. It may contribute to loss of PV+ cells by interfering with cell cycle regulation, proliferation, differentiation, migration and/or maturation^52^.

Loss of PV+ interneurons in *Aspm*^*1–7*^ mice may also relate to the observed ventriculomegaly and corpus callosum dysgenesis that resemble alterations seen in human microcephaly patients with *ASPM* mutations and in *Aspm*−/− mice^28–30^. As hypothesised by others, the *Aspm* mutation likely impairs ciliary function and precipitates lateral ventricle enlargement consequent to hydrocephalus^53^. The inverse correlation between enlarged ventricles and PV+ cell numbers as well as with IntelliCage® place learning and working memory suggests a connection. It is postulated that ASPM participates in cilia-mediated neuronal migration^53^. The N-terminal domain of *Aspm* is part of the family of ASH (ASPM, SPD-2, Hydin) domains, present in proteins associated with cilia and flagella^53^. In addition, primary ciliary signalling is necessary for interneuronal connectivity^54^ and a function of ependymal cilia is to transmit the directional information imperative for cellular migration^55^. Thus, the region-specific reduction in PV+ cell number may relate to impaired migration during development concomitant to compromised ciliary function. The *Aspm*^*1–7*^ mutation leads also to decreases in adult hippocampal neurogenesis (DCX+ cell number) in young adult mice. While it warrants further enquiry, given the role of ASPM in cell division and Wnt signalling, it could also be that *Aspm*^*1–7*^ spurs a reduction in adult neurogenesis through altered proliferative activity^56,57^. Reduced adult hippocampal neurogenesis consequent to disrupted Wnt signalling in rats impairs object recognition^58^. In addition, adult neurogenesis ablation can enhance hippocampal-dependent working memory under certain conditions^59^. Thus, whether the adult neurogenesis deficit in *Aspm*^*1–7*^ mice contributes to the object recognition and working memory phenotype needs to be confirmed. Additional analysis of *Aspm*^*1–7*^ mutation-induced developmental effects during embryogenesis would also be necessary to determine the origins of the brain tissue alterations observed here.

In sum, this analysis of *Aspm*^*1–7*^ mice has unearthed a complex cognitive phenotype including mild recognition memory impairment and clearly enhanced spatial memory. This outcome is partially consistent with findings in human MCPH patients. While the memory modalities affected may not overlap, human MCPH patients with *ASPM* mutations also showed mixed cognitive symptoms with mildly impaired cognition (including working memory) and intact mnesic abilities^15^. Although not reported previously in MCPH patients (as, potentially, it was not characterised in detail so far), the enhanced spatial memory described here is also a proposed endophenotype of specific rare ASD-subtypes, a disorder often comorbid with microcephaly in general^60,61^. ASD is highly complex and heterogeneous, related to variation in multiple genes^62^. *ASPM* was not identified as a susceptibility gene for either ASD or schizophrenia in recent genome-wide association studies from European and North American cohorts (that largely excluded Asian and African cohorts)^63,64^. Nevertheless, it may be that *ASPM* mutations, when present in certain rare cases (e.g. see ref. ^6^), contribute to this specific ASD endophenotype and not the full gamut of typical ASD features such as social avoidance and repetitive behaviour. There are now moves to elucidate convergent signalling pathways affected in NDDs to understand genetic overlap, improve disease diagnosis and facilitate treatment strategies^65^. In that respect, it is remarkable that the *Aspm*^*1–7*^-induced enhanced spatial memory is therefore also reminiscent of that found in mice with genetic modifications in the Wnt signalling pathway^45,66^. As *Aspm* is a positive regulator of the canonical Wnt/β-catenin pathway it is possible that dysfunctional Wnt signalling could underlie some of the phenotypes described here. We therefore propose the hypothesis that a potential *Aspm*^*1–7*^-induced excitation/inhibition imbalance that may result from altered PV+ interneuron number and/or additional modifications in key brain regions consequent to altered Wnt signalling, underlies the cognitive endophenotype. While confirmation is needed, our findings are consistent with a convergence of NDD-related risk genes onto the Wnt neurodevelopmental pathway and the PV+ interneuron population^67–69^. This information therefore has important implications for understanding the genetic overlap and improved treatment of NDDs associated with ASPM. Furthermore, we propose spatial memory alterations as a measurable correlate of TRN circuit activity that may be a viable clinical biomarker to imply disruption of this circuit.

## Supplementary information

Supplementary information
