# Supplementary material for: A truncating Aspm allele leads to a complex cognitive phenotype and region-specific reductions in parvalbuminergic neurons
Source: Transl Psychiatry. 2020 Feb 13;10:66. doi: 10.1038/s41398-020-0686-0 (PMC7026184; doi:10.1038/s41398-020-0686-0)
Supplement: Supplementary file 1 — Supplementary information [file 41398_2020_686_MOESM1_ESM.pdf]

## Supplementary materials and methods

### Animals

Mice were kept on a 12/12-hour dark-light cycle (lights on at 7am) in a temperature (22 - 24°C) and humidity controlled (50 – 60%) environment and provided *ad libitum* standard chow and water. The *Aspm*$^{1-7}$ gene trap mutant mouse line was generated as described previously [1]. In brief, the line was generated from ES cells [AA0137, AspmGt (AA0137) Wtsi; vector pGT0lxr; obtained from the Sanger Institute Gene Trap Resource] by blastocyst injection and chimeras that had germ line transmission were crossed to C57BL/6JOlaHsd mice for more than 10 backcross generations. For the AA0137 ES cell line, the insertion site for the vector pGT01xr was determined to be in the intron between exon 7 and 8, 1386 nt downstream from the intron start site. RT-PCR and immunohistochemistry were used previously to establish the expression of the truncated ASPM protein tagged with lacZ [1]. All experiments involved the use of male homozygous mutant mice and their littermate control wild types.

### Analysis of the *Aspm*$^{1-7}$ mice

After 4 weeks of voluntary running wheel access, mice (WT n = 11, *Aspm*$^{1-7}$ n = 10, WT RW n = 10, *Aspm*$^{1-7}$ n = 10) were observed in open field, Y-maze, social discrimination, object recognition and IntelliCage® for analysis of place and reversal learning and working memory. Subsequently, all mice were sacrificed at the age of 60 weeks and perfusion-fixed brain tissue was processed immunohistochemically to assess parvalbumin (PV) + interneuron marker numbers and volume estimates in selected brain regions of sedentary mice. A subset of younger (27 weeks old) sedentary WT and *Aspm*$^{1-7}$ mice (n = 3-4) were sacrificed and adult hippocampal neurogenesis (doublecortin (DCX)+ cell number) was analysed at a time point that coincided with the start of the behaviour testing sequence.

### Voluntary running wheel activity assessment

The voluntary running wheel behavior of the mice was analysed as described previously [2]. Mice from both the *Aspm*$^{1-7}$ and WT groups were assigned to either a running wheel (RW) or sedentary control group. All mice, including the sedentary controls, were housed in Tecniplast® standard mouse cages. Cages from mice assigned to the running wheel groups were accommodated with a low profile angled running wheel with a wireless controlled activity counter to quantify circadian wheel-running activity (Wheel Manager Software, Med

Associates Inc., VT, and USA). Wheels were not removed prior to behavioral testing. The activity was indexed by the number of wheel revolutions. Total running wheel activity from the first 4 weeks was analysed as well as the activity per day during this time.

**Behavioral assays**

**Open Field**

The Open Field (OF) analysis was carried out as described previously [2]. The arena consisted of a transparent and infra-red light permeable acrylic test arena with a smooth floor (internal measurements: 45.5 x 45.5 x 39.5 cm). Illumination levels were set at approx. 150 lux in the corners and 200 lux in the middle of the test arena. Data were recorded and analysed using the ActiMot system (TSE, Bad Homburg, Germany).

**Y Maze**

Spontaneous alternations were assessed using the Y-Maze as reported in a foregoing study [3, 4]. The Y maze was composed of opaque light grey PVC and had 3 identical arms (30 x 5 x 15 cm) placed at 120° from each other; the illumination in the center of the maze was maintained at 100 lux. For each test, a mouse was placed at the end of one arm and allowed to move freely through the maze during a 5-minute session. Spontaneous alternations (defined as consecutive entries into all three arms without repetitions in overlapping triplet sets) were scored. Total arm entry numbers were collected over the 5 minutes. Spontaneous alternation performance percentage (SPAs %) is further defined as the ratio of actual (total alternations) to possible alternations (total number of triplets) x 100. Alternation behavior is a measure of spatial working memory. The percentage alternate arm returns (AARs %) were also calculated where the animal, after moving to a new arm, returns to the previously visited arm.

**Social Discrimination**

The Social Discrimination procedure was applied in a manner similar to that previously described [3, 5]. The test consists of two 4-min exposures of stimulus animals (ovariectomized 129Sv females) to the test animal in a fresh cage to which the test animal had been moved 2 h prior to testing. During the first exposure, the sample phase, we introduced one stimulus animal into the cage and allowed it to roam freely with the test animal. A trained observer with a hand-held computer recorded the amount of time that the test animal explores (sniffing

of the head and body, direct contact) the stimulus. This measure of the sample phase was used as an index of social investigation and social affinity. After a retention interval of 2h, this stimulus animal was re-exposed to the test animal together with an additional, previously not presented stimulus animal. A separate "familiar" and "unfamiliar" stimulus animal was assigned to each test animal. A trained observer with a hand-held computer again recorded the duration of investigatory behavior of the test animal towards the stimulus animals (familiar and unfamiliar) during this test phase. A social recognition index was calculated as time spent investigating the unfamiliar stimulus mouse / time spent investigating both the familiar and unfamiliar stimulus mouse.

**Short and long-term object recognition memory**

The object recognition procedure implemented has been described in detail [4]. In brief, mice were allowed to habituate to empty cages for 10 minutes once each day during the two days prior to the test. In the sample phase, two identical objects were presented in the cage for the mouse to explore three times for 5 minutes each with a 15-minute inter-trial interval. Three hours later, during the short-term recognition memory test phase, one of these objects with a novel object was presented to the mouse to explore for 5 minutes. Long-term recognition memory was then tested 24 hours later by presenting one of the original objects and another novel object for 5 minutes. The amount of time spent by the mouse investigating the objects in each phase was recorded. A recognition index was then calculated as the percentage time spent investigating the unfamiliar novel object/ (time spent investigating familiar+unfamiliar objects).

**Place and reversal learning and working memory in the IntelliCage®**

Place and reversal learning and working memory were assessed using the IntelliCage® (detailed procedure in [4]). The IntelliCage® (NewBehavior, TSE Systems GmbH, Bad Homburg, Germany) is an automated behavioral analysis apparatus for assessing individually RFID-tagged (Planet ID "White Label" (Planet ID GmbH, Essen, Germany) mice living in social groups. The plastic homecage in this case is relatively large (55 x 37.5 x 20.5 $cm^3$) where the four cage corners are operant conditioning chambers (15 x 15 x 21 $cm^3$). Up to 10 mice are housed within one cage and can access drinking water in the corners. Only one mouse can enter each corner at a time ("corner visit") and nosepoke through an opening to access water ("nosepoke"). With

this apparatus, the mice were assessed for place learning as an index of spatial reference memory, reversal learning to measure behavioral flexibility and patrolling ability as an index of working memory. Two separate cages were used for this experiment and mice were housed according to genotype. During the “free adaptation phase” (days 1-3), all motorized doors were open and mice had free access to all corners to drink water. During this 3 day period, spontaneous activity and exploratory indices (nosepokes, corner visits) were recorded. The number of corner visits, nosepokes and percentage visits with nosepokes were compared between genotypes during the first hour and 24 hours after introduction into the IntelliCage® as a measure of habituation to a novel environment. On day 4, there was a nosepoke adaptation period where mice learned to nosepoke for water. On days 5-8, the mice learned to access water in one of the IntelliCage® corners (“correct corner”). The corner assigned to each mouse was the least preferred corner during the adaptation phase and attempts to access water in the other three corners registered as an error (“incorrect nosepoke”). The percentage error rate (# incorrect nose pokes/total # nose pokes x 100) was then calculated and a decreased error rate signified enhanced spatial reference memory ability. On days 9-12, after the place-learning task, the assigned corner for each mouse switched to the opposite corner. The percentage error rate (as above for place learning) was calculated for each animal and indexed the behavioral flexibility of the mouse. On days 13-16, working memory of the mice was assessed in the patrolling task. The mice learned that the assigned corner would be the next adjacent corner once water access was successful. This assignment switched to the next corner in a clockwise direction and three yellow LED lights, situated at each door, illuminated when the mouse entered the correct corner. We calculated the percentage error rate for each animal as before during place and reversal learning.

**Tissue preparation**

Mice were euthanised at the age of 60 weeks and perfused transcardially with a solution of 4% paraformaldehyde (PFA) in 0.1M phosphate buffer. Fixed brains were extracted, post-fixed overnight in a solution of 4% PFA at 4°C and then transferred to a 30% (w/v) sucrose cryoprotectant solution until saturated. Brains were then sectioned in a rostro-caudal direction on a dry ice-cooled block with a sliding microtome (Leica, Bensheim) into 40 µm-thick coronal free-floating sections and stored at -20°C in a cryoprotectant solution containing 25% ethylene glycol and 25% glycerine in phosphate buffer. In keeping with the systematic

random sampling necessary for stereological analysis, a one-in-six series of sections was taken for analysis.

**Immunostaining**

For immunostaining of doublecortin (DCX) + and parvalbumin (PV) + cells an Avidin-Biotin Complex ABC method like that employed previously [2, 6] was used. In brief, tissue was rinsed in phosphate buffered saline (PBS) 0.1M, pH 7.5, three times for 10 minutes each, followed by quenching of endogenous peroxidase using 0.3 % hydrogen peroxide solution in PBS for 30 minutes at room temperature on a shaker. The tissue was then blocked using a solution of PBS with 0.24 % triton-X (PBS-T) and 10 % fetal bovine serum (FBS) for 1 hour and then incubated in primary antibody solutions (in PBS-T). Primary antibody incubations took place over night at 4°C with continuous agitation. On the second day, tissue was rinsed in PBS 0.1M, pH 7.5, three times for 10 minutes each, followed by blocking of the tissue in blocking solution (PBS-T + 10% FBS) for 30 minutes. The tissue was then incubated for 2 hours in appropriate secondary antibody diluted in PBS. Subsequent to three 10 minute rinses in PBS 0.1M, the tissue was incubated for 2 hours in ABC complex prepared according to manufacturers instructions (VECTASTAIN Elite ABC HRP Kit PK-6100, VECTOR LABORATORIES, INC., Burlingame, USA). After another three 10 minute rinses in PBS, the chromogenic step was initiated with 3, 3'-diaminobenzidine (DAB) as the chromogen. Tissue was rinsed three more times in PBS, mounted and dried on slides, dehydrated in graded alcohols, cleared in Xylol and coverslipped. A primary rabbit polyclonal anti-DCX antibody (1:1000, Catalog #: Ab18723, Abcam) was used in this protocol with a biotinylated goat anti-rabbit IgG (1:300; Biotin-SP AffiniPure Goat Anti-Rabbit IgG, Jackson ImmunoResearch Inc, USA) and a primary monoclonal mouse anti-PV antibody (1:1000, Catalog #: PV235, SWANT, Switzerland) was used with a biotinylated rabbit anti-mouse IgG (1:300, Biotin-SP AffiniPure Rabbit Anti-Mouse IgG, Jackson ImmunoResearch Inc, USA). Negative controls, with omission of the primary antibodies, revealed no positive staining.

**Unbiased stereological estimates of DCX+ and PV+ cell numbers and volumetrics**

DCX+ and PV+ cell numbers were estimated in specific regions of interest (ROIs) using the Stereo Investigator software system (StereoInvestigator, MBF Biosciences Inc.) on every sixth serial 40 µm coronal section with the Optical Fractionator probe. A Zeiss Axioplan2 microscope

was equipped with StereoInvestigator image analysis software (MicroBrightField Inc., Williston, VT, USA). DCX+ cells were estimated within the dorsal hippocampal dentate gyrus. PV+ cell numbers were estimated within the hippocampal Cornu Ammonis (CA) 1 and 2/3 regions, the thalamic reticular nucleus and anterior cingulate cortex (ACC). A preliminary analysis was performed for all ROIs to ascertain the optimal sampling conditions. Using the 10x objective, the ROIs were outlined using the software according to the stereotactic coordinates in the mouse brain atlas as follows: dorsal hippocampal dentate gyrus between -0.94 to -2.92 mm, CA1, 2, 3 between -1.34 to -3.16 mm (sampling grid: 100 µm, counting frame: 100 µm), TRN between -0.46 to -1.94 mm (sampling grid: 300 µm, counting frame: 100 µm) and ACC between 1.18 to -0.10 mm (sampling grid: 100 µm, counting frame: 100 µm) [7]. Total cell number (N) within the ROI was then estimated according to the following formula:

$$N = \sum Q^{-} \times (1/ssf) \times (1/asf) \times (1/tsf)$$

In this formula, $Q^-$ represents the counts, *ssf* is the section-sampling fraction, *asf* is the area-sampling fraction and *tsf* is the thickness sampling fraction [8]. All immuno-positive cells within the counting frame placed systematically within the sampling grid and within the contours were counted bilaterally by scanning through the tissue in the x-y plane using the software and viewed with a 20x objective. The Gundersen's coefficent of error (CE, m = 1) was used to determine the precision of the measures and counts with a CE below 0.1 were considered valid. DCX+ and PV+ cells were only counted when clear cell bodies were visible. 4 sections for ACC, 6 sections for TRN, 8 sections for DG DCX and 7 sections for CA1, 2, 3 PV were analysed. Volumetric analysis of selected ROIs was performed using the Stereoinvestigator software and the Cavalieri estimator probe as described previously [6]. The following brain regions of interest were analysed: corpus callosum between 1.10 and -0.46 mm (5 sections, sampling grid: 15 µm), lateral ventricles between 1.18 and -1.06 mm (6 sections, sampling grid: 50 µm), CA1, 2, 3 and DG between 0.94 and 3.16 mm (7 sections, sampling grid: 15 µm).

**Statistics**

Numerical analyses were performed using GraphPad Prism version 7.03 for Windows (GraphPad Software, La Jolla, California, USA, www.graphpad.com). For continuous data meeting the assumption of normality with the Shapiro-Wilk test, a 2-way ANOVA was performed to test genotype-exercise interaction effects (All 2-way ANOVA results are shown in Supplemental Table 1 for Open Field, Y maze, object recognition and social discrimination).

When significant interactions were detected, a post-hoc Tukey's test was used to determine differences between groups. For tests with repeated measures (IntelliCage®), we used repeated measures (RM) ANOVA with day respectively as within-subject factor and genotype as between-subject factor. A post-hoc Sidak's test was performed to assess differences between the genotypes on each day with p values adjusted for multiple comparisons. When two groups were compared (DCX+ and PV+ cell analysis, total running wheel distance during first 4 weeks, IntelliCage® habituation phase nosepokes, corner visits and % visits with nosepokes) an unpaired Student's t-test was used. To assess whether a potential relationship exists between the behavioral and neuroanatomical alterations, we used a Pearson's correlation analysis to produce a correlation matrix for the cognitive, PV+ cell number and volumetric parameters altered in the *Aspm*$^{1\text{-}7}$ mice. A Grubb's test detected and excluded data outliers (one sedentary homozygous mutant mouse was identified as an outlier and excluded from long-term object recognition memory analysis). For all tests, a P value $< 0.05$ was considered significant and data are presented as means ± SEM. A correction for multiple testing of the various parameters was not performed.

# Supplementary Table 1. Behavior results and statistical analysis

## 2-way ANOVA results with genotype and exercise as factors

| | *Sedentary* | | *Exercise* | | *Genotype* | | | *Exercise* | | | *Genotype x Exercise* | | |
|---|---|---|---|---|---|---|---|---|---|---|---|---|---|
| | *WT Mean ± SEM* | *Aspm$^{1-7}$ Mean ± SEM* | *WT Mean ± SEM* | *Aspm$^{1-7}$ Mean ± SEM* | *F* | *df* | *p* | *F* | *df* | *p* | *F* | *df* | *p* |
| **Open field** | | | | | | | | | | | | | |
| **Distance moved total (cm)** | 21192.86 ± 870.57 | 20650.87 ± 579.41 | 23278.38 ± 1312.34 | 19089.98 ± 558.29 | 7.14 | 1,37 | 0.01* | 0.09 | 1,37 | 0.77 | 4.24 | 1,37 | 0.047* |
| **Speed (cm/s)** | 19.00 ± 0.87 | 18.52 ± 0.50 | 20.72 ± 1.19 | 16.95 ± 0.55 | 6.56 | 1,37 | 0.02* | 0.008 | 1,37 | 0.93 | 3.93 | 1,37 | 0.06 |
| **Rearing (#)** | 135.64 ± 4.59 | 142.60 ± 4.60 | 148.30 ± 10.59 | 114.90 ± 7.05 | 3.52 | 1,37 | 0.07 | 1.14 | 1,37 | 0.30 | 8.21 | 1,37 | 0.007** |
| **Centre time (%)** | 33.84 ± 2.64 | 29.53 ± 1.92 | 30.50 ± 2.86 | 31.78 ± 4.10 | 0.26 | 1,37 | 0.61 | 0.03 | 1,37 | 0.86 | 0.88 | 1,37 | 0.35 |
| **Y maze** | | | | | | | | | | | | | |
| **Spontaneous alternations (%)** | 66.54 ± 3.01 | 64.36 ± 4.11 | 62.46 ± 2.31 | 64.32 ± 4.02 | 0.002 | 1,37 | 0.96 | 0.36 | 1,37 | 0.55 | 0.35 | 1,37 | 0.56 |
| **Alternate arm returns (%)** | 30.09 ± 2.53 | 32.66 ± 5.36 | 32.61 ± 3.16 | 30.32 ± 3.28 | 0.001 | 1,37 | 0.97 | 0.00 | 1,37 | 0.98 | 0.43 | 1,37 | 0.514 |
| **Entries #** | 21.46 ± 2.05 | 19.50 ± 2.12 | 25.60 ± 2.99 | 17.40 ± 1.54 | 5.18 | 1,37 | 0.03* | 0.21 | 1,37 | 0.65 | 1.96 | 1,37 | 1.17 |
| **Social discrimination** | | | | | | | | | | | | | |
| **Recognition index** | 0.57 ± 0.03 | 0.58 ± 0.04 | 0.55 ± 0.04 | 0.60 ± 0.03 | 0.84 | 1,36 | 0.37 | 0.006 | 1,36 | 0.94 | 0.52 | 1,36 | 0.47 |
| **Investigation time (s)** | 16.20 ± 0.65 | 19.24 ± 1.76 | 19.05 ± 2.37 | 19.05 ± 1.75 | 0.79 | 1,37 | 0.38 | 0.61 | 1,37 | 0.44 | 0.79 | 1,37 | 0.38 |
| **Object recognition** | | | | | | | | | | | | | |
| **Index duration 3h** | 0.61 ± 0.04 | 0.48 ± 0.03 | 0.59 ± 0.04 | 0.53 ± 0.05 | 5.94 | 1,36 | 0.02* | 0.14 | 1,36 | 0.71 | 0.74 | 1,36 | 0.40 |
| **Index duration 24h** | 0.67 ± 0.06 | 0.57 ± 0.06 | 0.71 ± 0.06 | 0.62 ± 0.03 | 4.57 | 1,35 | 0.04* | 1.30 | 1,35 | 0.26 | 0.03 | 1,35 | 0.86 |

**Supplementary Table 2. Behavior results and statistical analysis**

Student's t-tests between genotypes

| | *Sedentary* | | *Exercise* | | *Genotype* | | |
|---|---|---|---|---|---|---|---|
| | *WT*<br>*Mean ± SEM* | *Aspm^{1-7}*<br>*Mean ± SEM* | *WT*<br>*Mean ± SEM* | *Aspm^{1-7}*<br>*Mean ± SEM* | *t* | *df* | *p* |
| **Exercise** | | | | | | | |
| **Running wheel activity (rev)** | | | 294622 ± 89297 | 230012 ± 72264 | 0.56 | 18 | 0.58 |
| **Intellicage** | | | | | | | |
| **Habituation hour 1 visits #** | 16.70 ± 2.41 | 7.50 ± 2.29 | | | 2.55 | 14 | 0.02* |
| **Habituation hour 1 nosepokes** | 16.70 ± 3.35 | 3.33 ± 1.33 | | | 2.97 | 14 | 0.01* |
| **Habituation hour 1 % visits nosepokes** | 53.64 ± 10.19 | 22.25 ± 5.27 | | | 2.25 | 14 | 0.04* |

## Supplementary Table 3. Histological analysis and statistical results

| | | | Genotype | | |
|---|---|---|---|---|---|
| | *WT* *Mean ± SEM* | *Aspm*$^{1-7}$ *Mean ± SEM* | *t* | *df* | *p* |
| **Parvalbumin (PV) + interneuron number estimates** | | | | | |
| **Thalamic reticular nucleus (TRN) #** | 62642 ± 2600 | 48157 ± 3051 | 3.52 | 14 | 0.003** |
| **Cornu Ammonis (CA) 1 #** | 2077 ± 168.1 | 1129 ± 153.1 | 3.92 | 13 | 0.002** |
| **CA 2/3 #** | 1210 ± 128 | 808.5 ± 81.56 | 2.34 | 13 | 0.036* |
| **Dentate gyrus (DG) #** | 469.2 ± 201.3 | 468.7 ± 215.8 | 0.004 | 10 | 0.997 |
| **Anterior cingulate cortex (ACC) #** | 11597 ± 942.9 | 9486 ± 730.5 | 1.65 | 10 | 0.131 |
| **Adult hippocampal neurogenesis** | | | | | |
| **Doublecortin (DCX) + cell #** | 9503 ± 79 | 3061 ± 368.4 | 8.13 | 5 | 0.0005*** |
| **Regional volumetric analysis** | | | | | |
| **Lateral ventricles mm**$^3$ | 1.752 ± 0.17 | 2.777 ± 0.27 | 3.46 | 14 | 0.004** |
| **Corpus callosum mm**$^3$ | 2.161 ± 0.09 | 1.737 ± 0.07 | 3.33 | 11 | 0.007** |
| **CA1 mm**$^3$ | 2.088 ± 0.04 | 1.939 ± 0.14 | 1.31 | 12 | 0.215 |
| **CA 2/3 mm**$^3$ | 1.744 ± 0.12 | 1.741 ± 0.17 | 0.02 | 12 | 0.987 |
| **Dentate gyrus mm**$^3$ | 0.3596 ± 0.01 | 0.365 ± 0.03 | 0.19 | 12 | 0.85 |

**Supplementary Table 4. Correlation matrix of altered parameters**

| Variables | OR 3h | OR 24 h | IC PL D1 | IC PL D2 | IC PL D3 | IC PL D4 | IC PAT D1 | IC PAT D2 | IC PAT D3 | IC PAT D4 | TRN PV+ # | CA1 PV+ # | CA2/3 PV+ # | LV vol | CC vol |
|---|---|---|---|---|---|---|---|---|---|---|---|---|---|---|---|
| **OR 3h** | - | | | | | | | | | | | | | | |
| **OR 24h** | .453$^{T}$ | - | | | | | | | | | | | | | |
| **IC PL D1** | .391 | .152 | - | | | | | | | | | | | | |
| **IC PL D2** | **.560*** | .133 | **.811***** | - | | | | | | | | | | | |
| **IC PL D3** | .476$^{T}$ | .015 | **.661*** | **.907******* | - | | | | | | | | | | |
| **IC PL D4** | .482$^{T}$ | .099 | **.630*** | **.847***** | **.854***** | - | | | | | | | | | |
| **IC PAT D1** | .188 | .294 | **.684*** | .449 | .372 | .517$^{T}$ | - | | | | | | | | |
| **IC PAT D2** | .236 | .514$^{T}$ | **.605*** | .432 | .515$^{T}$ | .454 | .547$^{T}$ | - | | | | | | | |
| **IC PAT D3** | .236 | .279 | **.735**** | .524$^{T}$ | **.561*** | .514$^{T}$ | .533$^{T}$ | **.912******* | - | | | | | | |
| **IC PAT D4** | -.187 | **.562*** | **.637*** | **.568*** | **.560*** | .485$^{T}$ | .540$^{T}$ | **.877******* | .874$^{T}$ | - | | | | | |
| **TRN PV+ #** | **.603*** | **.666**** | .495$^{T}$ | **.695**** | **.603*** | .541$^{T}$ | .334 | .459 | .413 | **.652*** | - | | | | |
| **CA1 PV+ #** | **.565*** | **.634*** | .400 | .366 | .329 | .494 | .526$^{T}$ | .503$^{T}$ | .402 | .558$^{T}$ | **.695**** | - | | | |
| **CA2/3 PV+ #** | .386 | **.551*** | .292 | .341 | .337 | .424 | .301 | .411 | .316 | .459 | **.687**** | **.815***** | - | | |
| **LV vol** | -.303 | -.023 | **-.674*** | **-.649*** | **-.718**** | -.541$^{T}$ | **-.554*** | **-.619*** | **-.614*** | **-.698**** | **-.583*** | **-.636*** | **-.709**** | - | |
| **CC vol** | .090 | .426 | .582 | .408 | .358 | .542 | .584$^{T}$ | **.654*** | **.620*** | **.660*** | **.582*** | **.776**** | **.818**** | **-.699*** | - |

*Correlation matrix of correlations between cognitive behaviour, volumetric and parvalbuminergic parameters altered in Aspm$^{1-7}$ mice: Pairwise correlations performed (N = 10-16 per group depending on analysis). T = statistical tendency, p < 0.10, *p < 0.05, **p < 0.01, ***p < 0.001, ****p < 0.0001, OR = object recognition, IC = Intellicage®, PL = place learning, D = day, PAT = patrolling, LV vol = lateral ventricle volume, CC vol = corpus callosum volume, PV+ = parvalbumin positive, CA = Cornu Ammonis, TRN = thalamic reticular nucleus. Green highlights = significant positive correlation, red highlights = significant negative correlation*

**References**


1. Pulvers, J.N., et al., *Mutations in mouse Aspm (abnormal spindle-like microcephaly associated) cause not only microcephaly but also major defects in the germline.* Proc Natl Acad Sci U S A, 2010. **107**(38): p. 16595-600.
2. Garrett, L., et al., *Voluntary wheel running in mice increases the rate of neurogenesis without affecting anxiety-related behaviour in single tests.* BMC Neurosci, 2012. **13**: p. 61.
3. Garrett, L., et al., *Fgf9 (Y162C) Mutation Alters Information Processing and Social Memory in Mice.* Mol Neurobiol, 2018. **55**(6): p. 4580-4595.
4. Holter, S.M., et al., *Assessing Cognition in Mice.* Curr Protoc Mouse Biol, 2015. **5**(4): p. 331-58.
5. Salminen, A.V., et al., *Meis1: effects on motor phenotypes and the sensorimotor system in mice.* Dis Model Mech, 2017. **10**(8): p. 981-991.
6. Garrett, L., et al., *Analysis of Neuropsychiatric Disease-Related Functional Neuroanatomical Markers in Mice.* Curr Protoc Mouse Biol, 2018. **8**(1): p. 79-128.
7. Franklin, K.B.J., G. Paxinos, and G.S.F. Rudolph, *The mouse brain in stereotaxic coordinates*. 2001, San Diego, Calif.; London: Academic.
8. West, M.J., L. Slomianka, and H.J. Gundersen, *Unbiased stereological estimation of the total number of neurons in thesubdivisions of the rat hippocampus using the optical fractionator.* Anat Rec, 1991. **231**(4): p. 482-97.
